# Benzyl 3-(2-methyl­phen­yl)dithio­carbazate

**DOI:** 10.1107/S160053681201567X

**Published:** 2012-04-18

**Authors:** Shahedeh Tayamon, Thahira Begum S. A. Ravoof, Mohamed Ibrahim Mohamed Tahir, Karen A. Crouse, Edward R. T. Tiekink

**Affiliations:** a Department of Chemistry, Universiti Putra Malaysia, 43400 Serdang, Malaysia; b Department of Chemistry, University of Malaya, 50603 Kuala Lumpur, Malaysia

## Abstract

In the title compound, C_15_H_16_N_2_S_2_, the central C_2_N_2_S_2_ unit is essentially planar (r.m.s. deviation = 0.047 Å) and forms dihedral angles of 68.26 (4) and 65.99 (4)° with the phenyl and benzene rings, respectively, indicating a twisted mol­ecule. Supra­molecular chains with a step topology and propagating along [100] feature in the crystal packing, mediated through N—H⋯S hydrogen bonds. The chains are consolidated into a three-dimensional architecture by C—H⋯π inter­actions.

## Related literature

For background to the coordination chemistry and bio-activity of hydrazinecarbodithio­ates, see: Khoo *et al.* (2005 ▶); Chan *et al.* (2008 ▶); Ravoof *et al.* (2010 ▶). For related structures, see: Paulus *et al.* (2011 ▶); Manan *et al.* (2012 ▶). For the synthesis, see: Tarafder *et al.* (2002 ▶).
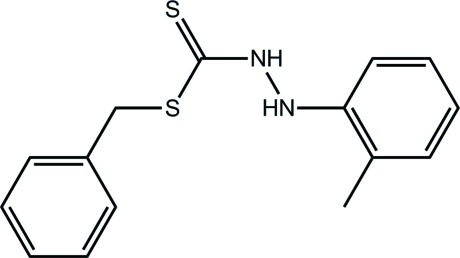


## Experimental

### 

#### Crystal data

C_15_H_16_N_2_S_2_
*M* *_r_* = 288.42
Monoclinic, 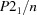

*a* = 5.7000 (1) Å
*b* = 11.0136 (2) Å
*c* = 22.7545 (4) Å
β = 95.198 (2)°
*V* = 1422.60 (4) Å^3^
*Z* = 4
Cu *K*α radiation
μ = 3.27 mm^−1^
*T* = 100 K
0.22 × 0.14 × 0.08 mm

#### Data collection

Oxford Diffraction Xcaliber Eos Gemini diffractometer
Absorption correction: multi-scan (*CrysAlis PRO*; Agilent, 2011 ▶) *T*_min_ = 0.63, *T*_max_ = 0.77
15011 measured reflections
2725 independent reflections
2560 reflections with *I* > 2σ(*I*)
*R*_int_ = 0.022

#### Refinement

*R*[*F* ^2^ > 2σ(*F* ^2^)] = 0.035
*wR*(*F* ^2^) = 0.097
*S* = 1.03
2725 reflections
179 parameters
2 restraints
H atoms treated by a mixture of independent and constrained refinement
Δρ_max_ = 0.44 e Å^−3^
Δρ_min_ = −0.23 e Å^−3^

### 

Data collection: *CrysAlis PRO* (Agilent, 2011 ▶); cell refinement: *CrysAlis PRO*; data reduction: *CrysAlis PRO*; program(s) used to solve structure: *SHELXS97* (Sheldrick, 2008 ▶); program(s) used to refine structure: *SHELXL97* (Sheldrick, 2008 ▶); molecular graphics: *ORTEP-3* (Farrugia, 1997 ▶) and *DIAMOND* (Brandenburg, 2006 ▶); software used to prepare material for publication: *publCIF* (Westrip, 2010 ▶).

## Supplementary Material

Crystal structure: contains datablock(s) global, I. DOI: 10.1107/S160053681201567X/hg5209sup1.cif

Structure factors: contains datablock(s) I. DOI: 10.1107/S160053681201567X/hg5209Isup2.hkl

Supplementary material file. DOI: 10.1107/S160053681201567X/hg5209Isup3.cml

Additional supplementary materials:  crystallographic information; 3D view; checkCIF report

## Figures and Tables

**Table 1:** Hydrogen-bond geometry (Å, °) *Cg*1 and *Cg*2 are the centroids of the C2–C7 and C9–C14 rings, respectively.

*D*—H⋯*A*	*D*—H	H⋯*A*	*D*⋯*A*	*D*—H⋯*A*
N1—H1*n*⋯S2^i^	0.88 (1)	2.50 (1)	3.3625 (14)	169 (2)
N2—H2*n*⋯S2^ii^	0.88 (1)	2.79 (2)	3.5919 (13)	152 (1)
C11—H11⋯*Cg*1^iii^	0.95	2.98	3.8594 (18)	155
C5—H5⋯*Cg*2^iv^	0.95	2.84	3.7005 (18)	151

Symmetry codes: (i) 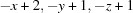; (ii) 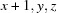; (iii) 
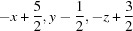
; (iv) 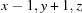.

**Fig. 1.**
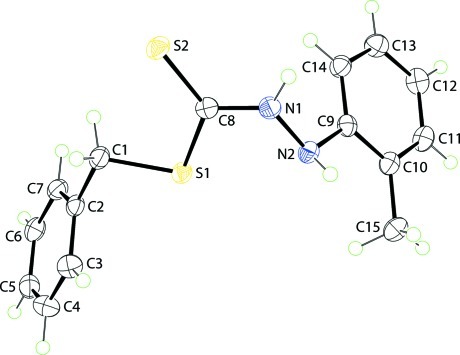
The molecular structure of (I) showing the atom-labelling scheme and displacement ellipsoids at the 50% probability level.

**Fig. 2.**
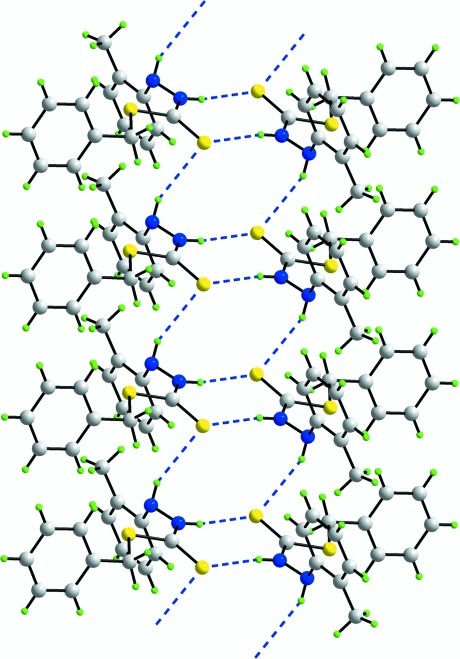
A view of the supramolecular chain in (I) mediated by N—H···S hydrogen bonding, shown as blue dashed lines.

**Fig. 3.**
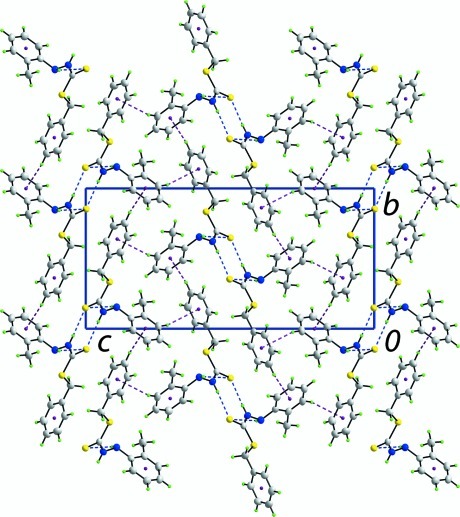
A view in projection down the a axis of the unit-cell contents for (I). The N—H···S hydrogen bonding and C—H···π interactions are shown as blue and purple dashed lines, respectively.

## supplementary crystallographic information

### Comment

The study of hydrazinecarbodithioate derivatives, their coordination chemistry and bio-activities has been carried out in our laboratory for a number of years (Khoo *et al.* 2005; Chan *et al.* 2008; Ravoof *et al.*, 2010; Paulus *et al.*, 2011; Manan *et al.*, 2012). In our efforts to expand the scope of these investigations, the title compound (I) was synthesized and characterized crystallographically.

In (I), Fig. 1, the central moiety is essentially planar with a r.m.s. deviation of the fitted atoms (S1,S2,N1,N2,C1 and C8) of 0.047 Å with the maximum deviations being 0.0709 (8) and -0.0711 (11) Å for the N2 and N1 atoms, respectively. The phenyl and *o*-tolyl rings lie out of this plane forming dihedral angles of 68.26 (4) and 65.99 (4)°, respectively. The *o*-tolyl ring is orientated to one side of the central plane as seen in the C8—N1—N2—C9 torsion angle of 111.24 (16)°, whereas the plane through the central atoms almost bisects the phenyl ring with the C8—S1—C1—C2 torsion angle being 176.89 (11)°.

The most prominent feature of the crystal packing is the formation of supramolecular chains along [100]. These are mediated through N—H···S hydrogen bonds involving the thione-S2 atom, Table 1. Thus, centrosymmetrically related molecules are connected into dimeric aggregates *via* eight-membered {···HNCS}_2_ synthons. These are connected to translationally related dimeric aggregates *via* weaker N—H···S hydrogen bonds leading to 10-membered {···HNNH···S}_2_ synthons. The chain has a step topology and comprises alternating {···HNCS}_2_ and {···HNNH···S}_2_ synthons, Fig. 2. The chains are consolidated into a three-dimensional architecture by C—H···π interactions, Fig. 3 and Table 1.

### Experimental

The compound was prepared by adapting the synthetic procedure for *S*-benzyldithiocarbazate (Tarafder *et al.*, 2002). *o*-Tolylhydrazine hydrochloride (0.1 mol) in absolute ethanol (70 ml) was added to a solution of potassium hydroxide (0.1 mol) in absolute ethanol (40 ml). The mixture was cooled in an ice-salt bath and carbon disulfide (0.1 mol) was added drop-wise with constant stirring over one hour, during which a yellow-orange solution was formed. The temperature of reaction was kept below 278 K. Benzyl chloride (0.1 mol) was added drop-wise to the above solution with vigorous stirring while continuing to maintain the temperature below 278 K. The pale product was filtered off, dried briefly in a vacuum desiccator over anhydrous silica gel, recrystallized from absolute ethanol and kept in a freezer overnight. Dark-yellow crystals were grown from its ethanol solution. Yield 89%. *M*.pt: 392 K. Anal. Found (Calc.): C, 62.03 (62.46); H, 5.34 (5.59); N: 10.26 (9.71)%.

### Refinement

Carbon-bound H-atoms were placed in calculated positions (C—H = 0.95 to 0.99 Å) and were included in the refinement in the riding model approximation with *U*_iso_(H) set to 1.2 to 1.5*U*_equiv_(C). The amino H-atoms were refined with a distance restraint of N—H = 0.88±0.01 Å, and with *U*_iso_(H) = 1.2*U*_eq_(N).

### Crystal data

**Table:**

C_15_H_16_N_2_S_2_	*F*(000) = 608
*M**_r_* = 288.42	*D*_x_ = 1.347 Mg m^−^^3^
Monoclinic, *P*2_1_/*n*	Cu *K*α radiation, λ = 1.54180 Å
Hall symbol: -P 2yn	Cell parameters from 8863 reflections
*a* = 5.7000 (1) Å	θ = 4–71°
*b* = 11.0136 (2) Å	µ = 3.27 mm^−^^1^
*c* = 22.7545 (4) Å	*T* = 100 K
β = 95.198 (2)°	Prism, dark-yellow
*V* = 1422.60 (4) Å^3^	0.22 × 0.14 × 0.08 mm
*Z* = 4	

### Data collection

**Table:**

Oxford Diffraction Xcaliber Eos Gemini diffractometer	2725 independent reflections
Radiation source: fine-focus sealed tube	2560 reflections with *I* > 2σ(*I*)
Graphite monochromator	*R*_int_ = 0.022
Detector resolution: 16.1952 pixels mm^-1^	θ_max_ = 71.5°, θ_min_ = 3.9°
ω/2θ scans	*h* = −6→6
Absorption correction: multi-scan (*CrysAlis PRO*; Agilent, 2011)	*k* = −13→13
*T*_min_ = 0.63, *T*_max_ = 0.77	*l* = −27→25
15011 measured reflections	

### Refinement

**Table:**

Refinement on *F*^2^	Primary atom site location: structure-invariant direct methods
Least-squares matrix: full	Secondary atom site location: difference Fourier map
*R*[*F*^2^ > 2σ(*F*^2^)] = 0.035	Hydrogen site location: inferred from neighbouring sites
*wR*(*F*^2^) = 0.097	H atoms treated by a mixture of independent and constrained refinement
*S* = 1.03	*w* = 1/[σ^2^(*F*_o_^2^) + (0.0646*P*)^2^ + 0.6201*P*] where *P* = (*F*_o_^2^ + 2*F*_c_^2^)/3
2725 reflections	(Δ/σ)_max_ = 0.001
179 parameters	Δρ_max_ = 0.44 e Å^−^^3^
2 restraints	Δρ_min_ = −0.23 e Å^−^^3^

### Special details

**Table:**

Geometry. All s.u.'s (except the s.u. in the dihedral angle between two l.s. planes) are estimated using the full covariance matrix. The cell s.u.'s are taken into account individually in the estimation of s.u.'s in distances, angles and torsion angles; correlations between s.u.'s in cell parameters are only used when they are defined by crystal symmetry. An approximate (isotropic) treatment of cell s.u.'s is used for estimating s.u.'s involving l.s. planes.
Refinement. Refinement of *F*^2^ against ALL reflections. The weighted *R*-factor *wR* and goodness of fit *S* are based on *F*^2^, conventional *R*-factors *R* are based on *F*, with *F* set to zero for negative *F*^2^. The threshold expression of *F*^2^ > 2σ(*F*^2^) is used only for calculating *R*-factors(gt) *etc*. and is not relevant to the choice of reflections for refinement. *R*-factors based on *F*^2^ are statistically about twice as large as those based on *F*, and *R*- factors based on ALL data will be even larger.

### Fractional atomic coordinates and isotropic or equivalent isotropic displacement parameters (Å^2^)

**Table:**

	*x*	*y*	*z*	*U*_iso_*/*U*_eq_	
S1	0.96691 (6)	0.83305 (3)	0.582743 (16)	0.02154 (13)	
S2	0.73047 (7)	0.64766 (4)	0.498745 (16)	0.02497 (14)	
N1	1.1403 (2)	0.62113 (12)	0.56054 (6)	0.0234 (3)	
H1n	1.153 (3)	0.5503 (11)	0.5436 (8)	0.028*	
N2	1.3086 (2)	0.65256 (12)	0.60680 (6)	0.0226 (3)	
H2n	1.446 (2)	0.6572 (17)	0.5924 (8)	0.027*	
C1	0.6928 (3)	0.90389 (14)	0.55250 (7)	0.0247 (3)	
H1A	0.6944	0.9153	0.5094	0.030*	
H1B	0.5571	0.8516	0.5597	0.030*	
C2	0.6713 (3)	1.02493 (14)	0.58237 (6)	0.0216 (3)	
C3	0.8217 (3)	1.12038 (16)	0.57225 (7)	0.0277 (4)	
H3	0.9444	1.1086	0.5472	0.033*	
C4	0.7947 (3)	1.23226 (16)	0.59816 (8)	0.0314 (4)	
H4	0.8989	1.2967	0.5908	0.038*	
C5	0.6166 (3)	1.25144 (15)	0.63490 (7)	0.0285 (4)	
H5	0.5978	1.3288	0.6524	0.034*	
C6	0.4669 (3)	1.15700 (15)	0.64578 (8)	0.0279 (4)	
H6	0.3445	1.1693	0.6709	0.033*	
C7	0.4950 (3)	1.04384 (15)	0.62004 (7)	0.0252 (3)	
H7	0.3931	0.9789	0.6282	0.030*	
C8	0.9545 (3)	0.69201 (14)	0.54650 (6)	0.0206 (3)	
C9	1.3110 (3)	0.57463 (14)	0.65724 (7)	0.0211 (3)	
C10	1.4934 (3)	0.59097 (14)	0.70252 (7)	0.0231 (3)	
C11	1.4978 (3)	0.51509 (15)	0.75165 (7)	0.0272 (3)	
H11	1.6205	0.5243	0.7825	0.033*	
C12	1.3278 (3)	0.42639 (16)	0.75675 (7)	0.0294 (4)	
H12	1.3361	0.3745	0.7902	0.035*	
C13	1.1459 (3)	0.41438 (16)	0.71253 (7)	0.0288 (4)	
H13	1.0267	0.3553	0.7161	0.035*	
C14	1.1371 (3)	0.48843 (15)	0.66296 (7)	0.0250 (3)	
H14	1.0114	0.4800	0.6328	0.030*	
C15	1.6766 (3)	0.68749 (16)	0.69763 (7)	0.0288 (4)	
H15A	1.7773	0.6925	0.7348	0.043*	
H15B	1.5991	0.7659	0.6894	0.043*	
H15C	1.7730	0.6670	0.6655	0.043*	

### Atomic displacement parameters (Å^2^)

**Table:**

	*U*^11^	*U*^22^	*U*^33^	*U*^12^	*U*^13^	*U*^23^
S1	0.0220 (2)	0.0224 (2)	0.0195 (2)	0.00052 (13)	−0.00217 (14)	−0.00249 (13)
S2	0.0232 (2)	0.0274 (2)	0.0232 (2)	−0.00015 (14)	−0.00370 (15)	−0.00584 (14)
N1	0.0266 (7)	0.0216 (6)	0.0210 (6)	0.0004 (5)	−0.0032 (5)	−0.0029 (5)
N2	0.0217 (7)	0.0245 (7)	0.0207 (7)	−0.0005 (5)	−0.0028 (5)	0.0004 (5)
C1	0.0217 (8)	0.0261 (8)	0.0253 (8)	0.0019 (6)	−0.0035 (6)	−0.0019 (6)
C2	0.0209 (7)	0.0242 (8)	0.0188 (7)	0.0017 (6)	−0.0030 (5)	0.0008 (6)
C3	0.0288 (8)	0.0312 (8)	0.0241 (8)	−0.0025 (7)	0.0065 (6)	−0.0002 (6)
C4	0.0372 (10)	0.0252 (8)	0.0323 (9)	−0.0060 (7)	0.0055 (7)	0.0007 (7)
C5	0.0320 (9)	0.0256 (8)	0.0268 (8)	0.0048 (6)	−0.0037 (6)	−0.0036 (6)
C6	0.0214 (8)	0.0359 (9)	0.0263 (8)	0.0047 (6)	0.0019 (6)	−0.0026 (7)
C7	0.0207 (7)	0.0287 (8)	0.0260 (8)	−0.0021 (6)	0.0003 (6)	0.0016 (6)
C8	0.0230 (7)	0.0233 (7)	0.0157 (7)	−0.0025 (6)	0.0026 (5)	0.0008 (6)
C9	0.0217 (7)	0.0214 (7)	0.0203 (7)	0.0037 (6)	0.0030 (6)	−0.0018 (6)
C10	0.0218 (8)	0.0269 (8)	0.0208 (7)	0.0033 (6)	0.0024 (6)	−0.0049 (6)
C11	0.0255 (8)	0.0366 (9)	0.0192 (7)	0.0045 (7)	0.0000 (6)	−0.0025 (6)
C12	0.0343 (9)	0.0339 (9)	0.0205 (8)	0.0048 (7)	0.0062 (6)	0.0034 (6)
C13	0.0283 (8)	0.0298 (8)	0.0289 (8)	−0.0017 (7)	0.0067 (6)	0.0010 (7)
C14	0.0234 (8)	0.0263 (8)	0.0247 (8)	0.0004 (6)	−0.0004 (6)	−0.0019 (6)
C15	0.0249 (8)	0.0355 (9)	0.0254 (8)	−0.0024 (7)	−0.0016 (6)	−0.0024 (7)

### Geometric parameters (Å, º)

**Table:**

S1—C8	1.7571 (15)	C5—H5	0.9500
S1—C1	1.8247 (16)	C6—C7	1.392 (2)
S2—C8	1.6729 (15)	C6—H6	0.9500
N1—C8	1.331 (2)	C7—H7	0.9500
N1—N2	1.4020 (17)	C9—C14	1.387 (2)
N1—H1n	0.877 (9)	C9—C10	1.407 (2)
N2—C9	1.432 (2)	C10—C11	1.394 (2)
N2—H2n	0.878 (9)	C10—C15	1.501 (2)
C1—C2	1.506 (2)	C11—C12	1.388 (2)
C1—H1A	0.9900	C11—H11	0.9500
C1—H1B	0.9900	C12—C13	1.384 (2)
C2—C3	1.389 (2)	C12—H12	0.9500
C2—C7	1.394 (2)	C13—C14	1.389 (2)
C3—C4	1.381 (2)	C13—H13	0.9500
C3—H3	0.9500	C14—H14	0.9500
C4—C5	1.389 (3)	C15—H15A	0.9800
C4—H4	0.9500	C15—H15B	0.9800
C5—C6	1.382 (2)	C15—H15C	0.9800
			
C8—S1—C1	101.79 (7)	C2—C7—H7	119.7
C8—N1—N2	120.74 (13)	C6—C7—H7	119.7
C8—N1—H1n	120.8 (13)	N1—C8—S2	121.91 (12)
N2—N1—H1n	118.2 (13)	N1—C8—S1	114.11 (11)
N1—N2—C9	114.19 (12)	S2—C8—S1	123.98 (9)
N1—N2—H2n	108.0 (13)	C14—C9—C10	120.37 (14)
C9—N2—H2n	112.9 (13)	C14—C9—N2	122.02 (14)
C2—C1—S1	108.06 (10)	C10—C9—N2	117.57 (14)
C2—C1—H1A	110.1	C11—C10—C9	117.97 (15)
S1—C1—H1A	110.1	C11—C10—C15	121.44 (14)
C2—C1—H1B	110.1	C9—C10—C15	120.59 (14)
S1—C1—H1B	110.1	C12—C11—C10	121.78 (15)
H1A—C1—H1B	108.4	C12—C11—H11	119.1
C3—C2—C7	118.62 (15)	C10—C11—H11	119.1
C3—C2—C1	121.22 (14)	C13—C12—C11	119.28 (15)
C7—C2—C1	120.15 (14)	C13—C12—H12	120.4
C4—C3—C2	120.69 (15)	C11—C12—H12	120.4
C4—C3—H3	119.7	C12—C13—C14	120.25 (16)
C2—C3—H3	119.7	C12—C13—H13	119.9
C3—C4—C5	120.58 (16)	C14—C13—H13	119.9
C3—C4—H4	119.7	C9—C14—C13	120.29 (15)
C5—C4—H4	119.7	C9—C14—H14	119.9
C6—C5—C4	119.37 (15)	C13—C14—H14	119.9
C6—C5—H5	120.3	C10—C15—H15A	109.5
C4—C5—H5	120.3	C10—C15—H15B	109.5
C5—C6—C7	120.12 (16)	H15A—C15—H15B	109.5
C5—C6—H6	119.9	C10—C15—H15C	109.5
C7—C6—H6	119.9	H15A—C15—H15C	109.5
C2—C7—C6	120.61 (15)	H15B—C15—H15C	109.5
			
C8—N1—N2—C9	111.24 (16)	C1—S1—C8—S2	−1.24 (12)
C8—S1—C1—C2	176.89 (11)	N1—N2—C9—C14	−9.7 (2)
S1—C1—C2—C3	68.74 (16)	N1—N2—C9—C10	172.43 (13)
S1—C1—C2—C7	−112.65 (14)	C14—C9—C10—C11	2.4 (2)
C7—C2—C3—C4	−1.1 (2)	N2—C9—C10—C11	−179.66 (14)
C1—C2—C3—C4	177.55 (15)	C14—C9—C10—C15	−177.54 (14)
C2—C3—C4—C5	0.0 (3)	N2—C9—C10—C15	0.4 (2)
C3—C4—C5—C6	0.5 (3)	C9—C10—C11—C12	−0.6 (2)
C4—C5—C6—C7	0.0 (2)	C15—C10—C11—C12	179.38 (15)
C3—C2—C7—C6	1.6 (2)	C10—C11—C12—C13	−1.4 (2)
C1—C2—C7—C6	−177.07 (14)	C11—C12—C13—C14	1.5 (2)
C5—C6—C7—C2	−1.0 (2)	C10—C9—C14—C13	−2.3 (2)
N2—N1—C8—S2	−170.63 (11)	N2—C9—C14—C13	179.88 (14)
N2—N1—C8—S1	8.92 (19)	C12—C13—C14—C9	0.3 (2)
C1—S1—C8—N1	179.22 (12)		

### Hydrogen-bond geometry (Å, º)

Cg1 and Cg2 are the centroids of the C2–C7 and C9–C14 rings, respectively.

**Table:**

*D*—H···*A*	*D*—H	H···*A*	*D*···*A*	*D*—H···*A*
N1—H1*n*···S2^i^	0.88 (1)	2.50 (1)	3.3625 (14)	169 (2)
N2—H2*n*···S2^ii^	0.88 (1)	2.79 (2)	3.5919 (13)	152 (1)
C11—H11···*Cg*1^iii^	0.95	2.98	3.8594 (18)	155
C5—H5···*Cg*2^iv^	0.95	2.84	3.7005 (18)	151

Symmetry codes: (i) −*x*+2, −*y*+1, −*z*+1; (ii) *x*+1, *y*, *z*; (iii) −*x*+5/2, *y*−1/2, −*z*+3/2; (iv) *x*−1, *y*+1, *z*.
